# First Language Attrition: What It Is, What It Isn’t, and What It Can Be

**DOI:** 10.3389/fnhum.2021.686388

**Published:** 2021-09-07

**Authors:** Federico Gallo, Beatriz Bermudez-Margaretto, Yury Shtyrov, Jubin Abutalebi, Hamutal Kreiner, Tamara Chitaya, Anna Petrova, Andriy Myachykov

**Affiliations:** ^1^Centre for Cognition and Decision Making, Institute for Cognitive Neuroscience, HSE University, Russian Federation, Moscow, Russia; ^2^Centre for Neurolinguistics and Psycholinguistics (CNPL), Vita-Salute San Raffaele University, Milan, Italy; ^3^Center of Functionally Integrative Neuroscience (CFIN), Department of Clinical Medicine, Aarhus University, Aarhus, Denmark; ^4^Department of Behavioral Sciences, Linguistic Cognition Laboratory, Ruppin Academic Center, Emek Hefer, Israel; ^5^Department of Psychology, Northumbria University, Newcastle upon Tyne, United Kingdom

**Keywords:** first language attrition, bilingualism, cross-linguistic interactions, EEG/MEG, fMRI

## Abstract

This review aims at clarifying the concept of first language attrition by tracing its limits, identifying its phenomenological and contextual constraints, discussing controversies associated with its definition, and suggesting potential directions for future research. We start by reviewing different definitions of attrition as well as associated inconsistencies. We then discuss the underlying mechanisms of first language attrition and review available evidence supporting different background hypotheses. Finally, we attempt to provide the groundwork to build a unified theoretical framework allowing for generalizable results. To this end, we suggest the deployment of a rigorous neuroscientific approach, in search of neural markers of first language attrition in different linguistic domains, putting forward hypothetical experimental ways to identify attrition’s neural traces and formulating predictions for each of the proposed experimental paradigms.

## Introduction

We live in an increasingly globalized world whose one inalienable feature is continuously growing international migration. UN DESA data suggest that international migration continues to grow year-on-year: it doubled in the last 20 years to reach 260 million in 2017 (United Nations, 2018). In the United States alone, the international migrant population approaches 50 million people. An inevitable product of mass migration is bi- and multilingualism—it is estimated that more than half of the world’s population now speak two or more languages (Ansaldo et al., 2008).

One of the key features of bi- and multilingualism is the necessity for the speakers to manage the simultaneous processing of two or more distinct languages. The existing literature provides ample evidence that this leads to a constant interplay between the first and the second language (L1, L2) at the levels of phonology (Goldrick et al., 2014), lexicon (Malt et al., 2015), and grammar (Hartsuiker et al., 2004). Most importantly for the purposes of this paper, interactions between L1 and L2 are reciprocal: not only do specific features of L1 affect the use of L2 [e.g., Hamada and Koda, 2008; Ionin and Montrul, 2010; Rasier and Hiligsmann, 2007), but performance in the native language also changes under the influence of L2 (e.g., Gürel, 2004; Schmid and Jarvis, 2014; de Leeuw, 2017; Kasparian and Steinhauer, 2017)]. An important and relatively understudied aspect associated with the latter is known as native language attrition — the gradual decrease of native language performance that occurs with time and may be associated with increased use of L2, decreased use of L1 or both [for a review see Köpke et al. (2019)]. Importantly, despite the long-standing interest in this topic, this phenomenon remains largely understudied, leaving us with a rather hazy understanding of the mechanisms that drive it and the factors that modulate it. In this paper we address a salient lacuna in this field, namely the neurocognitive mechanisms underlying linguistic attrition.

Aiming at building a unified theoretical framework allowing for generalizable results, this paper proposes a rigorous neuroscientific approach. To this end, we begin with an attempt to clarify the conceptualization and terminology used to describe the attrition phenomena. Subsequently, we to trace its phenomenological and contextual constraints in order to understand its origins. We discuss hypothesized underlying mechanisms, and briefly review the evidence supporting them. Finally, the main section of this paper provides the groundwork for methodological adjustments designed to provide a more systematic examination of attrition, focusing on the search for neural markers of L1 attrition in different linguistic domains.

### Defining Language Attrition: What It Is, and What It Is Not

The first thing one may notice when addressing a relatively young and underexplored field like language attrition is an almost equal ratio between experimental studies and theoretical contributions. One of the consequences of language contact that has attracted increasing attention is L1 attrition. Investigations from different perspectives (e.g., linguistics, philosophy, neuroimaging) have resulted in a heterogeneous conceptualization of the phenomena with inconsistent terminology, described by Köpke (2004b) as a “terminological jungle.” In this first section, we briefly review the terminological inventory used to describe L1 attrition, examining different conceptualizations for the relevant terms. We then try to tackle the definition problem by providing a series of distinctions between similar yet different phenomena resulting from language contact.

One helpful way to define language attrition is to initially determine what attrition *is not* and then synthesize different attempts to define what attrition *is* or *may be*. Köpke (2004b) proposed a series of characteristics that help to shape a definition of the phenomenon. Most generally, attrition is not an intra-generational process, but rather an individual change. In this context, it is important to clarify the term “l*anguage shift*,” which may refer to processes that occur at the societal level (Dorian, 1982; Gardner-Chloros, 2001; Milroy, 2001) or at the individual level. The latter refers to individual change in L1 usage that can be associated with cultural and contextual processes, as a result of the individual’s adaptation to L2 culture and its presuppositions. For example, the conceptualization of emotions as passive states, expressed mostly by adjectives in English, versus emotions as actions, expressed mostly by verbs in Russian, may be described as language shift, if found in L1 Russian speakers in contact with L2 English (Schmid, 2011). Thus, some scholars view language shift as an important aspect of language loss. However, an alternative conceptualization views the notion of *loss* as a generic term incorporating both individual language shift and L1 attrition. According to this commonly adopted view (e.g., De Bot, 2000; Schmid, 2002; Köpke, 2004a; De Leeuw, 2008; Zaretsky and Bar-Shalom, 2010), language *shift* refers to a sociolinguistic aspect of usage, whereas language *attrition* indicates changes occurring at the cognitive/psycholinguistic level.

Additional characteristics proposed by Köpke (2004b) to outline what attrition is not refer to the relationship between language use and language performance. According to Köpke (2004b), language attrition affects not only the amount or frequency of language use, but the *linguistic performance* as such. Hence, infrequent use of the language is not *per se* sign of attrition, as long as such infrequent use remains intact in other aspects of performance, such as speed and accuracy of production and comprehension.

Finally, Köpke (2004b) stresses that Attrition is not *a pathological* process due to a neurological, psychiatric, or other deficit, such as dementia or post-injury aphasia. In relation to such age-related factors, it is important to consider the onset age of L1 loss. Individuals that abandon their native language environment before puberty seem likely to experience a more severe loss (e.g., Karayayla and Schmid, 2019) than those whose attrition onset occurred later in life. This may be related to maturational constraints imposed by puberty on language acquisition. Building on—and expanding—the concepts originally proposed within the Critical Period Hypothesis (Lenneberg, 1967), more recent accounts (e.g., Kuhl et al., 2005) postulate the existence of an optimal time window during individual development for acquiring L1 or L2, after which the attainment of native-like proficiency becomes more difficult (although some exceptions have been reported) due to a decrease in the neuroplastic potential of the human brain that begins during adolescence. When applied to native language attrition, this framework assumes that a deep erosion of the L1 system would be far more likely if attrition onset occurred before the end of adolescence [although this point is still under debate, for a review, see Bylund (2009) and Schmid and Köpke (2017)]. This hypothesis is supported by research that has investigated attrition in adopted children, who show fast, almost absolute, and irreversible attrition of their native language following very early and total severance from L1 use (Isurin, 2000; Nicoladis and Grabois, 2002; Pallier et al., 2003; Ventureyra et al., 2004). Thus, pre-adolescent L1 loss may reflect incomplete L1 acquisition rather than L1 attrition *per se* in its strict definition (e.g., Bolonyai, 2007). However, more recent research has shown that adopted populations maintain and activate specific L1 neural representations even after suppression of early contact with the native language and total absence of L1 exposure and conscious recollection (Pierce et al., 2014). Nonetheless, in the current paper, we focus on late (i.e., postpubescent) attriters in an attempt to disentangle attrition processes from incomplete language acquisition due to prepubescent onset of language erosion [for a review, see Schmid and Köpke (2017)].

Capitalizing on the abovementioned points, Köpke and Schmid (2004) worked out what is probably, up to the present day, the most commonly accepted definition of language attrition: “the non-pathological decrease in a language that had previously been acquired by an individual.” Attrition would hence reflect a situation whereby a speaker is losing proficiency in a language he or she previously mastered, not due to any brain degeneration or an age-related cognitive impairment but as a result of “a change in linguistic behavior due to a severance of the contact with the community in which the language is spoken” (Schmid, 2008, p.10).

These general definitional constraints set a good starting point for understanding attrition. To gain a better insight, it is important to briefly point out the major factors that modulate attrition. Thus, the existing literature points to two important factors that predict the nature and the severity of L1 attrition. First, the individual’s attitude toward their native language appears to affect the development of attrition more than other factors. A remarkable example is a study investigating attrition in German Jewish refugees who fled to Anglophone countries before World War II: the degree of negative attitude toward German, the “oppressor’s language,” was found to be the most influential factor for the severity of attrition (Schmid, 2002). Second, the characteristics of exposure to the native language have been implicated as an influencing factor. For example, high frequency of L1 use is generally associated with better language retention [e.g., Karayayla and Schmid, 2019; Schmid and Yilmaz, 2018; for a review, see Schmid and Köpke (2017); see also section “Exposure to L1: Quantity and Quality”].

Nonetheless, despite these hugely important efforts of many previous studies to conceptualize and actually define what attrition *is*, this question is far from being resolved. Thus, the idea of L1 disuse as a fundamental factor on attrition has been questioned by reported evidence of attrition signatures even in the case of short periods of immersion experience (or length of residency, LoR), which in turn favors the role of L2 exposure, L1-L2 interactivity and L2-induced L1-inhibition (Dussias, 2004; Dussias and Sagarra, 2007; Linck et al., 2009). Indeed, more recent debates have become focused on the influence of L2 exposure, thus going beyond the traditional consideration of attrition as a consequence of reduced L1 contact (e.g., Schmid, 2011) and highlighting the changeable L1-L2 dynamics that result in neuro-cognitive and, importantly, observable and quantifiable changes (Kasparian, 2015; Kasparian and Steinhauer, 2016; Kasparian et al., 2017). According to Kasparian and colleagues, L1 attrition should be conceptualized as a “less efficient L1 processing, increased L2-to-L1 influence and decreased L1-to-L2 influence (i.e., decreased L1 co-activation)” and “may include effects of increased attention, monitoring (second-thoughts) and motivation to perform well (self-consciousness)” (Kasparian and Steinhauer, 2017, p. 710). Thus, in this view, L2-influence is considered as a modulating factor in L1 attrition, but importantly, as not necessarily determinant [although see Schmid and Köpke (2017) for a more deterministic view]. Understanding the degree to which L1 exposure as well as the influence of L2 are determinant for the reduction of L1 proficiency may shed light on the origins of attrition.

## The Origin of Attrition: Cross-Linguistic Influence, Language-Internal Reorganization, or Lack of Exposure?

In line with the aforementioned debate, a key question that still remains unsolved regarding attrition relates to its causal mechanism(s). Does L1 attrition result from progressive disuse of the native language? Or is attrition “collateral damage” of the L2 acquisition, originating from cross-linguistic influence? In the next section, we review these two essential mechanisms and the evidence that supports them as implicated in driving attrition. The first mechanism hypothesized to cause L1 attrition is progressive disuse of this language. To the extent that L1 attrition is caused by disuse, it is theoretically plausible to expect L1 attrition even in the absence of a newly acquired L2. Such a process may be underpinned by reshaping of the corresponding neurolinguistic circuits. The second mechanism proposed to cause language attrition is cross-language processes. To the extent that L1 attrition is modulated by cross-language interference, the nature and level of attrition can be expected to reflect the interaction between the two languages in terms of code-switching and cross -language resemblance. Such cross-language interference may be underpinned by patterns of brain activation that reflect co-activation of two language systems.

### L2 Effects Versus L1 Reorganization

The existing literature offers numerous attempts at assessing the role of L2 in L1 attrition; these studies, however, report mixed results. Several studies have demonstrated that various aspects of L2 knowledge and use may determine the depth of L1 attrition. These L2 effects have been reported in different linguistic domains, i.e., phonology (e.g., De Leeuw et al., 2018), morphology (e.g., Dussias, 2004), syntax (e.g., Chamorro et al., 2016a), lexicon (e.g., Schmid and Jarvis, 2014) and semantics (e.g., Ben Rafael, 2001), across different L1-L2 combinations, both in linguistically/typologically close pairs (e.g., German–Dutch; Ribbert and Kuiken, 2010) and in distant ones (e.g., Korean–French; Ventureyra et al., 2004). Overall, the process of attrition is typically attributed to cross-linguistic influence exerted by the L2 on the native language system (e.g., Ben Rafael, 2001; Hutz, 2004). For instance, Altenberg (1991) reported a study using a grammaticality judgment task, in which sentences that were ungrammatical in L1 (German) but marginal in L2 (English) were perceived as more acceptable than sentences completely ungrammatical in both languages. This result supports the L2-to-L1 cross-linguistic influence hypothesis, although it needs to be considered with caution, as this study used only two participants. In a much larger sample of Greek/English attriters, Pelc (2001) reported similar results in favor of L2-to-L1 transfer as the cause of L1 attrition. The results showed an influence of L2 grammaticality on the acceptability judgments of L1 ungrammatical sentences. In a more recent study, Kasparian and Steinhauer (2017) also reported findings indicative of L1-L2 crosslinguistic interplay as a causal factor for attrition. In their study on relative clause processing in Italian vs. English, the authors found that attriters, contrary to monolinguals, provided significantly lower acceptability ratings for relative clause constructions that were ungrammatical in their L2, English, although less preferred but grammatical in their L1, Italian.

However, the explanations offered by many of such studies are not necessarily unequivocal with respect to effects resulting from L2 influence. For example, the vast majority of attrition studies have examined bilinguals with English as L2. English is an *analytical* language, relying more on a limited inventory of syntactic choices than on lexical morphology and word order to render the underlying conceptual message. Given these features, when English is the L2, it is often argued that such effects would be a consequence of *L1-internal* factors, such as simplification or generalization, but misinterpreted as L2-to-L1 transfer (Schmid, 2002; Köpke and Schmid, 2004). Simplification and generalization consist of a shift toward a simpler common linguistic pattern that is functional to both L1 and L2 (even if less typical in L1). This process could be driven by the tendency, intrinsic to our brain, to favor cost-efficient processes: to relieve the cognitive burden on the linguistic system, an attriter would unconsciously shift to a less costly, simpler construction common to both languages Such constructions often happen to belong to the L2 English system and the process could therefore be misinterpreted as L2-to-L1 transfer (Schmid, 2002; Köpke and Schmid, 2004). In this line are the findings of Isurin (2005) who investigated the L2 influence on word-order preferences in L1 Russian/L2 English attriters in two developmental studies: a longitudinal case study and a cross-sectional study. Both studies used a story retelling task in Russian and reported a shift from the use of the VSO word order, which is relatively frequent in L1 Russian, toward SVO, which is frequent in both languages but specifically less frequent in story retelling in Russian. This result appears to support the L2 interference hypothesis, suggesting that the strong preference for SVO in English affected the frequency of use of alternative word orders in Russian. However, SVO is not only acceptable in Russian story retelling, it is also the most frequent word order in Russian overall. That was taken as an argument for the observed shift as reflecting L1-internal generalization processes rather than transfer from L2, interpreting that participants merely shift to the word order shared by both languages and very frequent in both L1 and L2. In a similar example, results obtained in Russian/English attriters by Laleko (2007) were reported as supporting the role of L1-internal factors in driving the attrition process. When borrowing English words during Russian speech, attriters were found to develop a simplified pattern for gender assignment, as compared to the standard in Russian. Yet, such new gender-assignment strategy still followed a simplified phonological construction derived from Russian and not from English: words ending in a consonant were deemed to be masculine. Nonetheless, since the attrition process developed along the lines of an L1-internal pattern, the author interpreted the results as supporting the role of language-internal processes, rather than L2 transfer, in causing L1 attrition. Importantly, it must be noted that even when such simplification and generalization mechanisms are likely operating in attriters, these might indeed be the product of L2 influence on L1 processing, rather than an L1 reorganization acting in isolation. Nonetheless, L1-internal reorganization has very often been interpreted as an isolated process separate from L2, even if such linguistic organization is likely triggered by the use of an L2. Moreover, the idea that the cause of attrition is likely circumscribed to L1 processing itself rather than a consequence of L2 influence has received some support from studies comparing groups of attriters with the same L1 but different L2s, which failed to report effects indicative of L2 influence. Gunnewiek (1998), for example, investigated semantic L2 interference in Portuguese/Dutch and Portuguese/French attriters. The findings indicated very little L2 interference; hence, the evidence was inconclusive with regard to L2 influence on semantics in the L1 attrition process. Similarly, Köpke’s (1999) study could not present a strong case for the exclusivity of L2 effects on morpho-syntactic attrition in German/English and German/French bilinguals, as L2 influence did not appear to be the only source of L1 attrition, with L1 internal reorganization appearing to also affect the attrition process (contrary to the author’s original hypothesis).

Overall, the literature yields mixed positions on the causes of the L2 effects. While some consider these results to point toward L2-to-L1 cross-linguistic influence and hence to L2 exposure as the main cause of L1 attrition, others suggest that attrition could originate within the boundaries of the L1 linguistic system, thus highlighting the causal role of L1-internal reorganization processes. This debate is an example of how unclear the picture can appear in current attrition research: two contrasting hypotheses may be supported by the same pattern of results, depending on which underlying mechanism of attrition one accepts. The consideration that, rather than being mutually exclusive factors, both L1-internal processes and L2 effects might be at play is therefore becoming more often adopted among researchers, as introduced by De Bot (2002): “L1 attrition is both a decline of retrievability of declarative linguistic knowledge and deproceduralization of linguistic knowledge in L1, and an increase of competition with L2 knowledge.” Although the specific weight of L1 and L2 factors is still an open question and requires further and more specific experimental procedures, several studies have already indicated the modulation of attrition performance by factors corresponding to L1 exposure, LoR in the L2 environment and L2 proficiency levels (e.g., Kasparian and Steinhauer, 2017).

In the next section, we deepen the discussion on the role played by L1-related factors in the attrition process, exploring the possibility that L1 attrition might stem from reduced exposure to L1, with a focus on the potential effects of quality, beside quantity, of such exposure.

### Exposure to L1: Quantity and Quality

Many studies discussed in the previous section attribute attrition to the influence of the L2. Extensive research, however, has also posited that attrition could result from reduced exposure to and/or use of, L1: “Attrition is the result of long-term lack of stimulation” (Paradis, 2007, p. 125). These studies have addressed this possibility by investigating the effect of the amount of L1 contact on the attrition process. Nonetheless, as with L2 effects, the corresponding evidence is quite inconsistent and, importantly, tends to point toward the interrelation of factors related to both L1 and L2 as responsible for attrition (De Bot, 2002). On the one hand, several studies have reported higher levels of attrition in participants who had a progressively weaker contact with their L1 (De Bot et al., 1991; Köpke, 1999; Isurin, 2007; Opitz, 2013; Bergmann et al., 2016; Chamorro et al., 2016b; Kasparian et al., 2017; Schmid and Yilmaz, 2018; Karayayla and Schmid, 2019), supporting the idea that the amount of L1 contact predicts the severity of attrition. Note that these studies have used a variety of experimental methodologies, including EEG (Kasparian et al., 2017), eye-tracking (Chamorro et al., 2016b), and behavioral methods. In contrast, other studies have failed to find a reliable correlation between the amount of L1 exposure and the severity of L1 attrition (Jaspaert and Kroon, 1989; Altenberg, 1991; Grosjean and Py, 1991; Olshtain and Barzilay, 1991; Major, 1992; Ben Rafael, 2001; Jarvis, 2003; Schmid and Jarvis, 2014). Different explanations have been provided to account for this inconsistency. One plausible explanation lies with the intrinsic difficulty of measuring the amount of L1 exposure. Indeed, since L1 exposure decreases by default with increase in L2 exposure, it is it particularly difficult to isolate the specific contributions of these two concurrent processes, except when testing sequential bilinguals with equal exposure to L1 and L2 in a non-immigration setting. Furthermore, L1 exposure assessment relies largely on self-reports, which may in turn be influenced by subjective factors such as an individual’s attitudes toward their native language, a factor that has been reported as determinant in the development of L1 attrition (Schmid, 2002; Ben-Rafael and Schmid, 2007), and which could thus bias their self-assessed responses.

Another important factor is the *quality* of exposure to L1, which may affect attrition beyond the effect *of the quantity of exposure.* Indeed, several studies have shown a dissociation between the susceptibility of attrition to exposure in formal (i.e., professional) and in informal (i.e., family or friends) contexts. Speakers who maintain higher levels of L1 usage in formal contexts have shown lower levels of attrition, whereas individuals who use L1 mainly in informal contexts experienced higher attrition levels (Schmid, 2007; De Leeuw et al., 2010; Schmid and Dusseldorp, 2010; de Leeuw et al., 2012; Yilmaz and Schmid, 2012). Explanations for this pattern of results typically stress the contribution of bilingual code-switching to language attrition. Code-switching is the term applied to situations in which “speakers routinely interleave their languages in the course of a single utterance and adapt words from one of their languages in the context of the other” (Green and Abutalebi, 2013, p. 518). Thus, such findings suggest that the frequency of daily code-switching may have a more substantial contribution to L1 attrition than the general amount of exposure to L1. It is argued that code-switching contexts lead to co-activation of the two languages in a bilingual mind (Green, 2011), which, in turn, facilitates cross-linguistic interplay between L2 and L1 and thereby accelerates the attrition process related to cross-language influence (Grosjean and Py, 1991). Conversely, a bilingual speaker who mostly uses L1 in a context where code-switching is rare or discouraged (e.g., in more formal circumstances, such as at work) would experience less L1-L2 interchangeability and therefore their L1 performance will exhibit lower levels of attrition. Nonetheless, even if code-switching is reduced and hence less determinant in formal contexts, co-activation of both languages may still occur, thus leading to higher levels of L2 inhibition which would in turn reduce L1 automaticity (e.g., longer latency responses, more second guesses and more second-pass analyses). Considering these behavioral indices might effectively inform our knowledge of the activation of L2 and its inhibition during L1 performance (Kasparian and Steinhauer, 2017).

More evidence supporting the relevance of *quality* rather than *quantity* of L1 exposure for the attrition process comes from a study that investigated L1 attrition *without L2 acquisition* (Baladzhaeva and Laufer, 2018). This study is, to the best of our knowledge, the only investigation of its kind. The study analyzed L1 attrition of lexical retrieval, grammaticality judgments of collocations and future tense formation in a sample of Russian speakers who emigrated to Israel with no knowledge of Hebrew as L2, and compared them to a group of Russian/Hebrew immigrant speakers living in Israel as well as to a Russian monolingual group living in Russia. The authors found evidence for L1 attrition in both groups living in Israel, with the no-Hebrew group performing comparably to the Hebrew-speaking group but significantly worse than the monolingual controls in grammaticality judgments. The authors attributed such results to what they named “second-hand attrition”: the extensive contact with the bilingual attriter group caused the monolingual immigrant group to “pick up” L2 influence even without direct knowledge of L2. This result further supports the notion of a major contribution of the *quality* of L1 exposure to the attrition process, as these individuals experienced attrition without any modification in the *quantity* of contact with their L1. Moreover, these findings highlight the blurred boundaries and complex interrelation between L2 exposure and L1 reorganization, pointing to the challenge of disentangling the contribution of each factor.

Taken together, the accumulating evidence depicts a complex picture. On the one hand, the findings reviewed above highlight the key role of exposure to L1, and in particular the *quality* of exposure, in the process of attrition. On the other hand, many studies suggest that cross-language effects may also contribute. In this scenario, addressing the neurophysiological processes involved in language attrition could help disentangle the contribution of each of these factors. However, the neurobiological bases of attrition remain largely underexplored, with the exception of few recent studies by the groups of Monika Schmid (Schmid, 2011; Bergmann et al., 2015a) and Karsten Steinhauer (Kasparian and Steinhauer, 2016; Kasparian et al., 2017). Although these two research groups may provide somewhat contradictory findings, they agree in proposing the use of neuroimaging methods as the essential basis for a coherent framework of L1 attrition. In the next section, we review the main findings on the neurocognitive mechanisms of attrition and propose further steps to address the neural correlates of attrition in different linguistic domains.

## Building a Framework: In Search of the Neural Correlates of Attrition

Even more than a common theoretical framework, attrition research requires a methodological boost. The *Oxford Handbook of Language Attrition* (Schmid et al., 2019) recently dedicated several chapters to neuroimaging approaches (Rossi et al., 2019; Steinhauer and Kasparian, 2019). While providing a thorough overview of existing studies in the field, these chapters reiterate the need for more research that would provide better conceptualization of this phenomenon. As argued by these authors, the use of imaging techniques seems particularly important in the investigation of L1 attrition, since these techniques are able to highlight changes in language representation and processing even when there are no appreciable changes in behavioral measures, as already shown in L2 learners (McLaughlin et al., 2004). Thus, adding neurophysiological data to more traditional behavioral measurements may be particularly beneficial for determining subtle attrition patterns, for instance in individuals with short L2 exposure, as already demonstrated by Kasparian and colleagues in their samples of “late” attriters (Kasparian and Steinhauer, 2016, 2017; Kasparian et al., 2017). Thus, future attrition studies might consider the systematic use of high-resolution neuroimaging methods and sophisticated research paradigms which, in combination, might shed light on the underlying mechanisms of L1 attrition. In the next sections, we intend to offer a comprehensive compilation of best practices for future attrition research. We begin by discussing general methodological issues in the field of language attrition that should be systematically addressed, and the best strategies to solve them. Then we suggest potential ways to investigate the neural correlates of attrition in various linguistic subsystems in both production and comprehension domains.

### Common Practice in Attrition Research?

In view of the ongoing discussion reviewed above, suggesting that L1 attrition might be caused by an interaction of L1- and L2-related factors, a central issue is how we can effectively determine the specific contributions of each of these factors to elucidate those most determinant for the development of attrition (and thus crucial for its mitigation). In this section, we will briefly discuss methodological adjustments that may elucidate this issue. We begin with the factors assumed to have robust effects on attrition, namely, bilingual characteristics of the population examined, and the time elapsed from attrition onset.

A fundamental issue in investigating attrition is the language history and background of the population examined. Unfortunately, in this respect we are constrained in a “natural experiment” in which balanced controlled conditions of these factors do not exist [see Schmid (2013) for an overview]. Nevertheless, we can gain informative data by comparing bilingual populations of different characteristics. First, by comparing attriters samples with bilingual populations that are not assumed to be susceptible to language attrition such as (a) individuals who are formally learning the attriters’ L1 as a second language (henceforth, *attriter’s language learners*, AL-learners), deployed successfully as a control group in Kasparian and Steinhauer (2016), and (b) bilingual minority populations who did not migrate to a different language environment and who regularly split their time between two languages (e.g., Basque-Spanish). Processes related to cross-language interference can be expected in such populations as well as in attriters and may reveal relationships between L2 proficiency and L1 attrition. However, to the extent that such comparisons exhibit differences between attriters and the two other groups, these differences may be ascribed to attrition [see Steinhauer and Kasparian (2019), for future directions with respect to L1-L2 pairings and different bilingual groups].

Second, by comparing matching L1s but differing L2s, one may gain insight into the relative contributions to native language attrition of (i) L2-to-L1 cross-linguistic influence and (ii) L1-related factors, especially if systematic variations across L1-L2 pairs are introduced (e.g., completely comparable L1-L2, L1-L2 differing in the functioning of a particular construction, in the presence of a particular feature, etc.). Results that show comparable patterns across diverse L2s (all other variables being matched across samples) would provide evidence for the unique contribution of L1-related processes, as it is highly unlikely that different L2s, and thus different linguistic patterns, would produce the same effect on the same L1, as advocated by the L2 transfer hypothesis. Conversely, results that differ between samples—involving, for instance, modifications in differing language sub-systems or structures, depending on the L2—would provide evidence in support of the L2 transfer hypothesis, since each L2 would affect L1 in a particular way. Importantly, even if such studies fail to provide conclusive evidence (e.g., Gunnewiek, 1998; Köpke, 1999, see section “L2 Effects Versus L1 Reorganization”), they might still prove highly informative if a meticulous description of the bilingual background of the sample is included. In this sense, systematic examinations of sample characteristics in (a) studies that find evidence for attrition and (b) studies that do not find such evidence might shed light on the language background factors that modulate attrition.

An Additional factor that has a robust effect on attrition is the time elapsed from attrition onset. Hence, longitudinal studies of L1 attrition, as well as cross-sectional approaches, are imperative, for following the deterioration in L1 proficiency along with the increase of L2 experience. In this sense, multi-year follow-up studies carried out in migrant populations could provide valuable information to validate L1 disuse/L2 interference as causal mechanism of attrition. For instance, if signs of L1 attrition, obtained in comparison to monolingual controls, increase as a function of L2 use even in the case of active contact with L1 (through family and L1 communities) then the L2 interference hypothesis would be reinforced. Nonetheless, it is possible that the performance of L1 attriters decreases over time due to both increase of L2 experience and decrease of L1 contact (either at quantitative or qualitative level), suggesting the causes of L1 attrition by L1 disuse or, more likely, by an interplay between the two. Given the difficulty of obtaining well-controlled groups of L1 attriters varying in the amount of L1 contact, the use of tools which can account for the contribution of each factor (L1 contact/L2 use) over time and within the same L1 attrition sample could prove beneficial. Indeed, such procedures could mitigate the problems related to the obtaining of well-balanced control groups, a problem typically faced in the study of such a highly heterogeneous phenomenon as language attrition [see Schmid (2013), for an overview]. For instance, Steinhauer and Kasparian (2019) argue for the use of multiple regression methods and their application to longitudinal data as a beneficial approach in future attrition research.

Moreover, besides age of onset of L1-related processes, we should also take into consideration the role played by age of L2 acquisition. Indeed, depending on differences in this variable, we may encounter different attriter profiles, with some individuals acquiring their L2, and possibly reaching full bilingual attainment, *before* the onset of the attrition process and others for whom the start of L1 attrition and L2 acquisition are concomitant.

Finally, in view of the large variability involved in attrition it may be helpful to use a big-data approach. For example, harvesting posts from Facebook groups of attriters over several years and using big data methods of analysis might shed light on the time-course of attrition. That would provide valuable information regarding the modulation of specific L1 and L2 features, their interplay as well as the influence exerted by other extra-linguistic features (e.g., groups of social interaction, socioeconomic status, educational level, etc.).

Importantly, additional efforts should be aimed at developing methodological procedures that would be sensitive to subtle modifications in language use due to attrition. Existing research has failed to detect signs of attrition on several occasions (e.g., Jordens et al., 1989; De Bot and Clyne, 1994; Gunnewiek, 1998; Hulsen, 2000; Gürel, 2015; Karayayla and Schmid, 2019) or has reported minimal evidence of attrition (Altenberg, 1991; De Bot et al., 1991; Olshtain and Barzilay, 1991; Jaspaert and Kroon, 1992; Major, 1992; Köpke, 1999; Hutz, 2004) in samples where it was expected. These failures may be due to the use of research methods that are not sensitive enough, such as grammaticality judgments, or susceptible to social desirability, such as self-report questionnaires. Another reason might be that many of these studies addressed differences in accuracy, in which even attriters’ performance is often at ceiling level. This issue has been reviewed by Schmid (2013), who showed that error rates range between 1 and 5% (see e.g., Schmid, 2002, 2010; Montrul, 2008; Schmid and Dusseldorp, 2010; Stolberg and Münch, 2010). Conversely, reaction times might be a much more sensitive measure for capturing subtle differences in attriter performance, as suggested in previous studies showing longer latencies in attriter groups (e.g., Kasparian and Steinhauer, 2016, 2017). This pattern of results indicates the need to consider online processing and reaction time measures beyond accuracy indices when addressing attrition. Besides behavioral methods, recent studies [for a review see Rossi et al. (2019) and Steinhauer and Kasparian (2019)] suggest that systematic use of neuroimaging methods may provide the means to reveal subliminal signatures of attrition. These studies suggest that neurophysiological modifications might precede the appearance of overt behavioral changes and provide early indications of attrition when it is still behaviorally undetectable. As such methods provide highly detailed information about changes in brain activity in terms of timing, localization and connectivity, they may provide early markers of language attrition. Moreover, understanding the mapping between such neurophysiological changes and the differential characteristics of different groups of attriters and other bilingual speakers (e.g., bilingual minorities and AL-learners) may contribute to understanding the specific contribution of L1 and L2-related mechanisms to L1 attrition. Thus, even in the case of very subtle, or even undetectable behavioral modifications that can be associated with attrition, the findings of group differences between attriters and AL-learners or between different groups of attriters with different L2s would be informative about the underpinning of attrition.

Finally, language is a complex set of functions (e.g., production vs. comprehension; spoken vs. written) that operate on different levels of representations—phonology, morphology, lexicon, syntax, and pragmatics. The mixed results described above may be related to testing different functions at different levels of representation. Note that different functions are more susceptible to attrition than others, for example, production seems to be more susceptible to attrition than comprehension. In the same vein, different levels of representation may be differentially susceptible to attrition; for example, due to its very early age of acquisition, L1 phonology may be more resistant to L2 influence and hence less likely to show a pattern of attrition, whereas the lexicon and particularly entries with late age of acquisition (AoA) may be highly prone to attrition, due to cross-language interference [see Bardovi-Harlig and Stringer (2010) and Schmid and Köpke (2009) for reviews]. Research methods that could map the different effects of attrition (relative to either L1 or L2 factors) at different levels of representation (from minimal phoneme/grapheme units, to lexico-semantics or syntax) and for different language functions (i.e., production, comprehension) should provide a more comprehensive understanding of attrition.

Moreover, the experimental tasks deployed to evaluate attrition deserve more attention. As mentioned earlier, several studies have failed to reveal signs of attrition even when expected. This failure may be due to the very low error rates typically shown by attriters even when the attrition process is effectively underway, which potentially indicates that the tasks employed are not sensitive enough to tackle these processes. Increasing task complexity has indeed been shown to be an effective tool for discriminating slight variations from the norm. An example is the case of syntax comprehension during aging, for which age-related impairments emerge in the face of high task difficulty levels (e.g., Peelle et al., 2010; Antonenko et al., 2013), but not at low ones (e.g., Tyler et al., 2010). Therefore, we suggest that future attrition research could deploy linguistic tasks of various complexity levels, which might help to achieve better separation between the monolingual and the attriting samples. This has already been proven as an effective strategy in previous studies addressing syntactic attrition and using complex morphosyntactic manipulations, such as local- and non-local number agreement between inflected constituents (Kasparian and Steinhauer, 2016), syntactic violations embedded in relative clauses (Kasparian and Steinhauer, 2017) or relative clauses with high and low attachment (Dussias and Sagarra, 2007). The complexity of these tasks must guide future studies in the development of new paradigms sensitive enough to capture the subtly of L1 attrition.

### Investigating Neural Correlates of L1 Attrition

Up to this point, we have provided some general methodological considerations. We will proceed now to offer a collection of potential ways to investigate L1 attrition in different linguistic sub-systems. Following a brief literature overview, we will outline specific neuroimaging settings for each task in an attempt to isolate the neural correlates of the attrition phenomenon. Before we go into the different sub-systems of language ability, it is important to underline a common principle that we will follow when suggesting different tasks. L1 attrition research usually relies upon a native monolingual control sample. In this sense, when investigating the neural correlates of L1 attrition, native monolinguals’ brain response are taken as a baseline. Nonetheless, it would be advisable to include bilingual groups varying in dominance and proficiency, as well as to analyze the effect of key factors (e.g., exposure, proficiency and LoR) on the neural responses elicited from L1 processing; that would rule out the possibility that any differences found are actually merely a bilingualism effect obtained by a comparison to a monolingual sample. This is particularly important since the inclusion of a “pure monolingual” sample is linked to a limitation that cannot be easily avoided: since nowadays purely monolingual speakers are difficult to find, especially among young age groups, there is a high risk to classify as monolinguals individuals who are actually just native speakers of a target language, but who may still know (or be learning) other languages. This might introduce confounds related to neural and cognitive changes caused by L2 learning. Hence, including bilingual individuals distributed across the bilingual continuum constitutes an alternative solution.

Furthermore, a working strategy that future studies might consider in order to gain insight into the phenomenon of attrition is to reverse-engineer its nature from the results of second language acquisition studies, an idea first introduced in L2 attrition (Osterhout et al., 2019) and extensively discussed in recent L1 attrition research (Kasparian and Steinhauer, 2016, 2017). After all, multilingualism is indeed a continuum, a flexible experience: proficiency in each of our languages varies continuously depending on a series of factors, such as the amount of exposure to each language or the new ones we acquire. At one end of this spectrum, L2 acquisition may involve the process of accommodating an additional linguistic system in our brain. At the other end, attrition may entail the process of progressively losing access to one linguistic system [or, more realistically, some sub-components of it; see Kasparian and Steinhauer (2016), for extensive discussion on this continuum]. Thus, in this view, the process of L1 attrition in the brain should in some way mimic the reversal of proficiency gain in L2. If L1 attrition is characterized by a relative decline in L1 proficiency, then the difference between an L1 attriter and a monolingual speaker should at the neural level resemble/share similarities with the difference between a low proficient and a high proficient L2 learner. L1 performance/processing in attriters should also be associated with ERP and fMRI markers of reduced language proficiency, when compared to their L1 monolingual peers. In other words, in this logic L1 attriters’s ERP and fMRI profiles may be similar to that of bilinguals with lower L2 proficiency. Studies on bilingual language processing highlight that the timing and spacing of brain activity related to L2 processing tend to overlap with the patterns of activity elicited by L1 processing, as L2 proficiency increases. Conversely, as L2 proficiency decreases, more extensive brain activity, as well as reduced and delayed event-related potentials (ERP), are elicited by L2—compared to L1—processing [for reviews, see Abutalebi and Chang-Smith (2012) and Birdsong (2006)]. Hence, we will suggest tasks that should elicit differential brain responses between monolingual natives and L1 attriters, as well as predictions of ERP signatures and fMRI activation patterns one might expect to see in attriters both at production and language comprehension levels. By comparing patterns of brain activity between the putative attriting sample and the monolingual controls, on one hand, and the AL-learners, on the other, we envisage that one would be able to detect the effective presence or absence of L1 attrition across specific stages of the linguistic processing. Tables 1, 2 present a detailed list of predictions of attriters’ brain activity patterns during language production and comprehension, as compared with monolingual native speakers.

**TABLE 1:** Predictions of brain activity patterns during language production obtained by means of functional neuroimaging methods at corresponding anatomic-structure and dynamic-temporal levels.

Linguistic subsystem	Task and process involved	Brain activity pattern	
		Spatial localization (fMRI) (+) Activation intensity (+) Activation spread	Temporal dynamics (EEG/MEG) (−) Amplitude (+) Latency
Lexico-semantic	Picture naming task: Conceptual access and lexical retrieval processes	Left middle temporal gyrus Left Inferior frontal gyrus	∼100 ms (conceptual access)
			∼200 ms (P200, lexical selection)
Syntax	Scene description task, syntactic decision task: Syntactic encoding processes (selection of word class and grammatical gender, specification of words relations and inflectional marks)	Left inferior frontal gyrus Left rolandic operculum	∼400 ms (syntactic encoding)
Phonology	Picture naming task, picture-word interference paradigm: Phonological encoding and phonetic/articulatory preparation	Left inferior frontal gyrus Premotor cortex	∼300 ms (phonetic encoding) ∼450 ms (motor articulation, syllabification)

*Predictions are proposed for an attriter sample in comparison with their corresponding L1 monolingual controls. Note that, conversely, the attriter pattern would also resemble that obtained by a group of AL-learners.*

**TABLE 2:** Predictions of brain activity patterns during language comprehension obtained by means of functional neuroimaging methods at corresponding anatomic-structure and dynamic-temporal levels.

Linguistic subsystem	Task and process involved	Brain activity pattern	
		Spatial localization (fMRI) (+) Activation intensity (+) Activation spread	Temporal dynamics (EEG/MEG) (−) Amplitude (+) Latency
Phonology	Phoneme identification task, oddball paradigm: Recognition of phonological patterns	Left superior and medial temporal gyri Left inferior parietal lobule	∼150 ms (MMN, phonological discrimination)
Lexico-Semantic	Categorization/Judgment task, sentence congruency/semantic priming paradigm: Conceptual access, word prediction and integration	Left middle and superior temporal gyri	∼400 ms (N400, conceptual access)
Syntax	Grammatically Judgment task: Syntactic comprehension processes (building syntactic structure, thematic role assignment, integration and reanalysis processes)	Left inferior frontal gyrus Posterior superior temporal gyrus sulcus	∼150 ms (ELAN, building of syntactic structure) ∼300 ms (LAN, morphosyntactic integration) ∼400 ms (N400, thematic role assignment) ∼600 ms (P600, syntactic revision and reanalysis processes)

*Predictions are proposed for an attriter sample in comparison with their corresponding L1 monolingual controls. Note that, conversely, the attriter pattern would also resemble that obtained by a group of AL-learners.*

#### Lexico-Semantic L1 Attrition

It is widely accepted that the lexical system is one of the language domains most susceptible to attrition (e.g., Köpke, 2002; Schmid, 2011). Lexico-semantic attrition has been broadly documented in speech production—in the form of slow-downs in lexical retrieval as reflected in decreases in response accuracy (e.g., Olshtain and Barzilay, 1991; Stoessel, 2000; Schmid, 2009; Schmid and Jarvis, 2014), increased frequency and persistence of pauses, repetitions, hesitations, self-corrections (e.g., de Leeuw, 2007; Schmid and Fägersten, 2010; Yilmaz and Schmid, 2012; Schmid and Jarvis, 2014; Bergmann et al., 2015b) and Tip-of-the-Tongue experiences (Kreiner and Degani, 2015). L1 attrition has also been shown to manifest in impoverished lexical diversity (e.g., Laufer, 2003; Yilmaz and Schmid, 2012; Schmid and Jarvis, 2014). Lexico-semantic L1 attrition has been also found to be affected in the comprehension domain, e.g., with poor access to lexical representations during reading (Linck et al., 2009). One important and largely unresolved issue is whether lexico-semantic attrition effects only reflect changes in lexical access or spill into problems with semantic retrieval. Whereas some findings suggest that comprehension of semantic aspects is vulnerable in L1 attrition (Tsimpli et al., 2004), other studies failed to demonstrate L1 attrition in semantic processing, low sensitivity of paradigms being a potential cause (Scherag et al., 2004).

In regards to language production, different psycholinguistic models have postulated two main stages involved in the process of lexico-semantic access, namely, the *conceptual stage*, taking place from 100–150 ms, during which the semantic concept (i.e., the non-linguistic message) is prepared, and the *lexical stage*, taking place around 150–275 ms and involving the selection of the lemmas and the phonological form to be produced (Levelt, 1999; Indefrey and Levelt, 2004; Indefrey, 2011). Studies using ERP methodology have provided data supporting this view: whereas semantic effects have been found to modulate the brain signal at a very early time window (∼100 ms), reflecting fast access to the concept during speech production (Maess et al., 2002; Laganaro et al., 2009; Dell’Acqua et al., 2010), lexical selection has been registered in a positive waveform peaking later—starting from 200 ms (Laganaro et al., 2012; Valente et al., 2014). Concerning spatial patterns of activation, particular brain areas in the left temporal cortex have been found to underlie lexico-semantic retrieval, namely the anterior temporal cortex and the posterior middle temporal gyrus [see Indefrey and Levelt (2004, 2000) for reviews].

The most popular task for investigating the lexico-semantic system during speech production is the picture naming task, easy to perform using neuroimaging methods such as EEG/MEG and fMRI [see Indefrey and Levelt (2004, 2000) for detailed reviews on the task]. Studying the effect of semantic and lexical variables during the picture naming of attriters and control groups would allow us to determine the existence of L1 attrition at both conceptual and lexical stages of speech production. Thus, the cumulative semantic interference effect, namely increased effort in naming pictures that belong to the same semantic category of previously named pictures (Howard et al., 2006), would make it possible to identify the putative neural signatures of L1 attrition at the stage of conceptual retrieval, reflected in early (∼100 ms) ERP modulations, as reported in previous studies (e.g., Maess et al., 2002; den Hollander et al., 2019). Alternatively, manipulations of lexical frequency or the age of acquisition (AoA) of words included in the picture naming task might be used to tackle differences between attriters and controls in the stage of lexical retrieval reflected at later latencies (∼200 ms). Indeed, several ERP studies have shown the influence of these variables at this lexical stage, affecting the amplitude, latency or topography of the brain signal (e.g., Indefrey and Levelt, 2004; Costa et al., 2009; Dell’Acqua et al., 2010; Strijkers et al., 2010; Aristei et al., 2011; Laganaro and Perret, 2011; Valente et al., 2014). Similar manipulations would allow elucidation of delayed or impaired L1 lexical retrieval in attriters in comparison to control groups, as found between younger and older adult speakers (den Hollander et al., 2019) or between healthy controls and anomic patients (Laganaro et al., 2009). Comparisons of the intensity and localization of brain activation (e.g., using fMRI) or the timing and the latency of electrophysiological activity (in EEG) between the attriter sample and the two control groups would reflect the presence or the absence of attrition. A larger overlap of the attriters’ neural responses with the monolingual control sample would putatively suggest a lesser degree of attrition in the target group. Conversely, attriters’ patterns of neural activity similar to those exhibited by the group of AL-learners would support the presence of L1 lexical attrition. Importantly, further comparisons with bilinguals of various proficiency levels would provide a definitive proof of L1 attrition, together with the testing of different linguistic variables (e.g., exposure, proficiency) as key modulatory factors of attriters’ brain signal [see Kasparian and Steinhauer (2016) and Steinhauer and Kasparian (2019), for relevant ERP studies on lexical L1 attrition with monolinguals and AL-learners as control groups].

As for the comprehension domain, several EEG studies have demonstrated that lexico-semantic access takes place in a time window ranging from 200 to 400 ms post stimulus onset (Holcomb and Neville, 1991; Van Petten et al., 1999; Rodriguez-Fornells et al., 2002). This evidence has been systematically observed in the modulation of the N400, probably the most well-studied ERP component in relation to word processing, considered as a robust neural correlate of lexico-semantic processing (Kutas and Hillyard, 1980). Note, however, that earlier lexico-semantic effects have been also reported during word recognition (between 100 and 200 ms), denoting the cascaded-interactive nature of linguistic processing both in the visual (Penolazzi et al., 2007; Dikker and Pylkkanen, 2011) and the spoken domain [Pulvermüller et al., 2006; Van Den Brink and Hagoort, 2004; see Nieuwland (2019) and Pulvermüller et al. (2009) for reviews on early effects during language comprehension under different perspectives]. At neural localization, the access to word meaning has been supported by a temporo-frontal network of brain regions from the left posterior and middle temporal to left inferior frontal gyri [see Friederici (2012) for a review], areas which are, indeed, typically reported as the neural generators of the N400 effect (Van Petten and Luka, 2006; Lau et al., 2008).

Regarding the N400, this is a negative deflection starting around 200 ms and peaking at circa 400 ms following word onset, initially observed in response to words presented in semantically incongruent sentence conditions (Kutas and Hillyard, 1980), reflecting prediction processes during language comprehension (Van Petten and Luka, 2006; Lau et al., 2013). Besides semantic factors, the N400 amplitude has also been found to be influenced by lexical factors, such as the lexicality and the frequency of the words (Van Petten and Kutas, 1990; Van Petten, 1993; Kutas and Federmeier, 2000; Dambacher et al., 2006). In this sense, higher N400 amplitudes for semantically incongruent words as well as for meaningless and low-frequency words are considered to reflect the effort to predict, access and integrate the linguistic information into the preceding context [see Kutas et al. (2006) and Kutas and Federmeier (2011) for reviews]. N400 effects obtained under semantic priming paradigms, in which more positive-going N400 responses are obtained for words following a semantically related word than an unrelated one, are considered to reflect the facilitation in their lexico-semantic access triggered by the activation of the associated concept (Bentin et al., 1985; Deacon et al., 2000; Kiefer, 2002). Both predictability and semantic priming effects render the N400 component a cross-domain neural marker of the relative processing cost associated with lexico-semantic access, particularly useful for the analysis of the prediction and integration processes during word-meaning access. Importantly, the amplitude of the N400 component has also been found to be modulated as a function of L2 proficiency, reflecting facilitation in the lexico-semantic processing of L2 words due to the learning process (McLaughlin et al., 2004; Borovsky et al., 2010; Elgort et al., 2015).

The evidence reviewed above suggests that the N400 component might function as a neural marker for the attrition of the L1 lexico-semantic system in the comprehension domain. Specifically, opposite patterns of the N400 effect could be expected in attriters vs. highly proficient L2 learners. Òwo main paradigms should be considered when exploring the lexico-semantic system in L1 attriters, namely, sentence congruency and semantic priming paradigms, typically used to elicit the N400 (discussed above). In both, the presentation of L1 words under congruent/incongruent sentence conditions as well as under related/non-related semantic priming conditions could be expected to show smaller N400 effects for attriters and AL-learners than for monolinguals. Furthermore, word frequency effects on N400 would be also expected to differ between groups, with higher effort in the access to lexico-semantic representations of low frequency L1 words in attriters and AL-learners than in monolinguals. Such paradigms would make it possible to determine whether the attriter sample actually shows a lower capacity to effectively predict and integrate the incoming information into the preceding linguistic context as well as an increased effort during accessing lexico-semantic representations of L1 words, detecting an eventual malfunctioning of the lexico-semantic system during L1 language comprehension. Indeed, such effects have recently been reported by Kasparian and Steinhauer (2016): attriters with less frequent use of L1 showed reduced L1 activation during L2 processing, which can be interpreted as higher efficiency in L1 inhibition. Key paradigmatic features to be considered in further attrition studies would be the selection of L1 words associated with a higher processing difficulty and the use of both masked and unmasked priming paradigms [see Novitskiy et al. (2019) and Steinhauer et al. (2008) for ERP studies reporting cross-linguistic lexico-semantic effects through priming paradigms]. These features would enable analysis of lexico-semantic attrition, likely reflected in the differential modulation of N400 and P600 components, considering previous findings revealing attrition effects in both of these components (e.g., Kasparian and Steinhauer, 2016).

#### Phonological L1 Attrition

Attrition of the L1 phonological system has been documented both in language production—in the form of non-native-like pronunciation (e.g., Schmid, 2002; De Leeuw et al., 2010, 2018), and in comprehension –affecting the ability to distinguish between L1 phonemes (e.g., Ventureyra et al., 2004; Celata and Cancila, 2010) or to judge foreign accents in L1 (e.g., Major and Baptista, 2009).

Two main phonological processes in language production are considered to take place following the point of lexical selection, namely phonological encoding and phonetic/articulatory preparation. Research using monolingual samples consistently shows a time window between 275 and 450 ms for brain activity related to phonological encoding, which is followed by phonetic encoding and motor articulation processes in the 450–600 ms window (e.g., Eulitz et al., 2000; Indefrey and Levelt, 2004; Laganaro et al., 2009; Sahin et al., 2009; Dell’Acqua et al., 2010; Indefrey, 2011). Furthermore, fMRI studies have successfully isolated a left-lateralized network that supports phonological processing, involving the left posterior superior and middle temporal gyri, inferior parietal lobule and the left inferior frontal gyrus (e.g., Dehaene-Lambertz et al., 2002; Heim and Friederici, 2003; Indefrey and Levelt, 2004; Démonet et al., 2005; Indefrey, 2011).

The use of paradigms such as picture naming and picture-interference naming, in combination with EEG/MEG (Roelofs et al., 2016; Bürki, 2017) and fMRI (De Zubicaray et al., 2002) methods, have contributed to the understanding of the time course of word production and hence could provide valuable information about the neural markers of L1 attrition at the phonological and phonetic encoding stages. The logic is similar to the one described above for the evaluation of lexico-semantic L1 attrition. In the case of phonology, the manipulation of lexical and particularly phonological variables should be taken into account as well as the comparison of L1 attriters to both L1 monolinguals and AL-learners. For instance, analysis of the phonological facilitation ERP effects by means of a picture-word interference paradigm could allow us to identify specific neural patterns of phonological attrition in L1. Under this manipulation [see Mahon et al. (2007) for a review], participants are asked to name a picture which is superimposed with a phonologically/orthographically related word, which activates subsets of phonemes, including those in the target word, thus reducing naming latencies. This effect is considered to reflect facilitation of the phonological encoding of the noun, and has been found to modulate the ERP signal, starting around 300 ms post-stimulus onset (Dell’Acqua et al., 2010), although this facilitatory effect has also been reported in later time windows, starting from 450 ms after stimulus onset (Zhu et al., 2015). Accordingly, attriters would be expected to show a lower phonological facilitation effect in comparison to monolingual controls, that is, a reduced modulation of the ERP signal starting around 300 ms post-stimulus onset. This would be indicative of impaired encoding of phonological word forms in L1. A signature of attrition could also be revealed if comparable phonological facilitation effects were observed in attriters and AL-learners.

Phonological processing takes place earlier in language comprehension than in language production, potentially reflecting the reverse process of phonological access. Indeed, the recognition of phonemes during speech perception emerges in native listeners as early as around 50 ms following stimulus onset (Palva et al., 2002). This phonological process is often reflected in the modulation of the so-called mismatch negativity (MMN) effect. The MMN is an auditory event-related potential associated with general auditory perception and it has been specifically related to the brain’s capacity to automatically detect changes during auditory perception with minimal attention allocation [see Näätänen et al. (2007), for a review]. More specifically, MMN is defined as a negative potential which increases around 150–250 ms after the presentation of a deviant sound and whose neural generator is typically found in the auditory cortex (Kropotov et al., 1995, 2000; Rosburg et al., 2005). Several EEG/MEG studies have systematically reported a modulation of the MMN during the presentation of the standard-deviant stimuli (phonemes as well as of syllables and whole words) but, importantly, only in the listener’s native phonology (Dehaene-Lambertz, 1997; Näätänen et al., 1997; Shtyrov et al., 1998, 2000; Pulvermüller et al., 2001). In this sense, the MMN modulation is a neural correlate of the accuracy of the phonological system in recognizing native phonological patterns (Kirmse et al., 2008)—by means of language-specific sound memory traces formed through exposure to a native language during the first (6–12) months of life (Cheour et al., 1998; Kraus and Cheour, 2000; Hisagi et al., 2010). Interestingly, changes in the modulation of the MMN have also been shown to reflect acquisition of new phonological representations in the L2, with shifts from no initial MMN modulation to enhancements linked to increased L2 proficiency (Tremblay et al., 1998; Winkler et al., 1999; Cheour et al., 2002; Nenonen et al., 2005; Tamminen et al., 2015; Saloranta et al., 2020). For instance, it has been shown that L1 Hungarian/L2 Finnish speakers produce higher MMN modulation in response to Finnish phonemes than Hungarian monolinguals but similar to native speakers of Finnish (Winkler et al., 1999). This pattern suggests that the formation of phoneme representations of the foreign language is a consequence of repeated experience through learning. In this sense, and considering the previously proposed reverse-engineering hypothesis for L2 (e.g., Osterhout et al., 2019) as well as for L1 attrition (Kasparian and Steinhauer, 2016, 2017), a reverse pattern of results could be expected in L1 attrition if the ability to effectively discriminate native phonology is reduced in attriters.

We suggest that an appropriate task aimed at evaluating the presence of L1 attrition in phonological processing during speech comprehension would call for using the oddball paradigm. Recording EEG or MEG signals during the presentation of standard and deviant sounds (using L1 phonemes, syllables or words) and with increased differences between the two types of stimuli would provide useful information about the discrimination of phonemes in the native language. If the accuracy of attriters’ phonological system is reduced, then we would expect them to show a lower modulation of the MMN during the presentation of L1 stimuli than monolingual controls. In particular, higher stimulus deviation might be required to elicit the MMN in attriters, suggesting impoverished discrimination of phonetic patterns in the native language. As pointed out before, comparisons with bilinguals of various levels of proficiency as well as simultaneous bilinguals might be useful when determining different patterns of MMN modulation [see for instance Molnar et al. (2014) for discussion of simultaneous bilinguals as a more appropriate control group for detection of MMN differences]. Moreover, attriters would be expected to show performance similar to AL-learners, confirming the presence of phonological L1 attrition for auditory language comprehension.

We also propose the use of fMRI to investigate the brain regions involved in phonological L1 attrition during speech comprehension, following previous studies (Wang et al., 2003; Callan et al., 2004, 2014). For instance, Callan et al. (2004) investigated differential brain activation patterns related to processing of the/l/-/r/phonetic pair in native English speakers for whom the contrast is very easy to detect as well as in Japanese-English bilinguals, who lack the distinction in their native language. The stimuli were English syllables beginning with/r/or/l/(followed by different English vowels), vowels presented alone, and baseline trials consisting of silence. Participants had to identify whether the stimulus started with/l/,/r/, or a vowel. After a practice session outside the scanner, stimuli were presented in an fMRI scanner during acquisition in an event-related design. Callan and colleagues reported overlapping activation loci between the two groups for/r/and/l/phoneme identification; however, the Japanese native speakers showed significantly higher brain activation. The corresponding neural network included superior and medial temporal areas, Broca’s area, anterior insula, premotor cortex, cerebellum, and basal ganglia—regions known to be involved in phonetic processing, speech planning, and articulatory mapping. The additional activation reported for the non-native speakers is thought to underlie increased effort in L2 phoneme identification. Japanese participants also activated executive control areas known to be linked to inhibition of the interference from a dominant L1 during L2 processing in bilingual participants (see e.g., Green and Abutalebi, 2013). This study offers a useful model for assessing phonological L1 attrition as well as a representative baseline. A phonemic contrast on the L1/L2 pairs of the attriter sample and comparison of attriters’ pattern of brain activation with those of monolinguals and AL-learners would make it possible to test for the presence or absence of attrition.

Finally, we would like to add some considerations regarding the L1 attrition of orthographic processing in attriters. As opposed to the spoken domain, where signs of reduced discrimination for L1 phonemes have been well documented (Ventureyra et al., 2004; Major and Baptista, 2009; Celata and Cancila, 2010), the existence of comparable deficits in the processing of the basic units of the L1 orthographic system, namely graphemes, has rarely been addressed. Such deficits wouldn’t be surprising, given the tight interplay between phonological and orthographic processes, especially during early stages of visual word recognition, where phonological assembly plays a key role (Share and Stanovich, 1995; Perfetti, 2003; Kyte and Johnson, 2006). The study of orthographic L1 attrition, particularly putative deficits in grapheme-to-phoneme decoding during L1 reading, could be directly addressed from a neuroscientific point of view by using EEG/MEG, among other high temporal-resolution methods. In this sense, differences between attriters and control groups in the modulation of specific ERP components related to early stages of visual word recognition (first 200 ms of word processing), such as the P1/N1 complex or the P200 component, should be particularly addressed. Thus, differences obtained in these early brain responses would reflect deficits during the extraction of visual features and word-form orthographic analysis (Bentin et al., 1999; Assadollahi and Pulvermüller, 2001, 2003; Liu et al., 2003; Proverbio et al., 2004; Carreiras et al., 2005; Kutas et al., 2006; Coulson, 2007; Wu et al., 2012), thus confirming the deterioration of L1 orthographic processing. Conditions of particular interest would be those comparing the processing of words varying in script or writing system across attriter’s L1 and L2, thus exploring the degree of orthographic L1 attrition depending on the amount of consistency between L1-L2 decoding patterns.

#### L1 Attrition of Grammar

L1 attrition of grammar has previously been shown in the form of intrusion effects of the L2 on the L1 grammar as well as reductions and simplifications in the L1 morpho-syntactic system in both production and comprehension [e.g., Altenberg, 1991; Ben-Rafael, 2004; Gürel, 2004; Kasparian et al., 2017; Schmid, 2014; for a review, see Gürel (2008)].

Regarding production, it has been found that the encoding of syntax during speech production engages the activation of frontal as well as motor areas in sentence generation tasks, particularly the left inferior frontal gyrus (BA 44/45) and the left anterior part of Rolandic operculum (BA 6), caudally adjacent to Broca’s area (Indefrey et al., 2001; Haller et al., 2005; Tremblay and Small, 2011). A few electrophysiological studies have also contributed to the understanding of brain dynamics during syntax encoding, some of them suggesting these processes take place around 400 ms, once conceptual information has been activated, following a serial or cascaded processing during speech (Schmitt et al., 2001a, b).

However, syntactic processes have been much more studied in comprehension. Thus, there is general agreement that morphosyntactic processing during language comprehension is mediated by a fronto-parietal, left-lateralized brain network comprising the posterior portion of Broca’s Area (BA 44), and the posterior superior temporal gyrus/sulcus dorsally associated through the arcuate and the superior longitudinal fasciculi [for reviews, see Cappa (2012) and Friederici et al. (2006)]. Moreover, several EEG studies have identified a set of ERP components related to different stages of syntactic processing during language comprehension in both spoken (Friederici, 2002) and visual (Molinaro et al., 2011) perception modalities. For instance, violations of phrase structure have been found to elicit modulations in brain activity as early as 150–200 ms post stimulus onset, reflected in an Early Left Anterior Negativity (ELAN). This component has been related to the initial processing stage of syntactic structure building (Friederici, 2002; Herrmann et al., 2011; Fonteneau, 2013), although debate about the reliability of ELAN as a signature of syntactic analysis continues [for a review, see Steinhauer and Drury (2012)]. In comparison, Left Anterior Negativity (LAN) has been more systematically found at later latencies—around 300 ms—and it is related to difficulties with integrating morphosyntactic information during the subsequent stage of thematic role assignment (Friederici et al., 1993; Friederici and Frisch, 2000; Gunter et al., 2000; Molinaro et al., 2011). At the same time, the N400 component, previously reviewed in relation to lexico-semantic processes, has also emerged in relation to sentence-level violations, particularly difficulties in semantic integration [for a review, see Hahne and Friederici (2002)] and thematic role assignment (Frisch et al., 2004). Finally, the P600 component, a late centro-parietal positivity, has been linked to syntactic revision and reanalysis processes during the final stages of syntactic integration [e.g., Gouvea et al., 2010; for reviews see Friederici (2011) and Friederici and Weissenborn (2007)].

The timeline, chronometry, and neural generators of syntactic processing effects have been traditionally investigated by means of grammaticality judgment tasks and with the help of EEG/MEG or fMRI methods. In such tasks, participants are presented with grammatical and ungrammatical sentences that they have to rate for their acceptability. Importantly, the detection of grammatical violations in the non-native language has been reported to modulate both early and late syntax-related ERP components, showing a gradual acquisition of L2’s grammatical patterns (Rossi et al., 2006; Batterink and Neville, 2013; Tanner et al., 2013; Hanna et al., 2016). With regard to L1 attrition, a reverse-engineering approach might once again help us to determine whether L1 syntactic processing is affected in attriters and which specific neural sub-processes are at a disadvantage. This goal can be achieved by investigating differences and commonalities of ERP syntactic effects between attriters, on the one end, and monolinguals and AL-learners, on the other.

Very recent research in syntactic L1 attrition has provided initial examples of this ERP-comparison approach (Kasparian and Steinhauer, 2016, 2017; Kasparian et al., 2017; Miller and Rothman, 2020; Steinhauer and Kasparian, 2020). For instance, the study by Kasparian et al. (2017) investigated attrition of L1 grammar in a sample of Italian native speakers who emigrated to Canada in adulthood. Participants were presented with a grammaticality judgment task with simultaneous EEG recording. The study revealed differences in amplitude, scalp distribution, and duration of LAN, N400, and P600 components between the L1 Italian/L2 English attriter group and an Italian monolingual control group, providing evidence for L1 attrition at the neural level. Kasparian and colleagues’ approach represents a useful model to follow in L1 attrition research. Importantly, all their studies included a group of AL-learners, which allowed to obtain attritter neural patterns that followed a continuum modulated by L1 proficiency. Thus, those individuals with low L1 proficiency, either L1 attriters or AL-learners, showed no sensitivity to lexico-semantic violations caused by confusable words, as reflected in their reduced N400 responses. Conversely, L1 attriters and AL-learners with high L1 proficiency were found to be indistinguishable from L1 native controls in their sensitivity to such violations. These results demonstrate the importance of key control groups to investigate L1 attrition effectively.

We are unaware of any grammar attrition study using fMRI methodology; however, considerations and predictions similar to the ones already offered above can be applied. Existing fMRI studies show that syntactic processing relies upon a more extensive neural network in low proficient bilinguals compared to monolinguals, both in the form of increased activity in Broca’s area and in the activation of additional surrounding areas for the bilingual group only. Nonetheless, with increasing levels of L2 proficiency, the pattern of neural activation related to grammar processing in bilinguals has been shown to approximate that of monolinguals [for reviews, see Abutalebi (2008), Birdsong (2006) and Indefrey (2006)]. These results from the bilingualism literature enable us to make predictions about putative neural signatures of grammatical processes in L1 attrition (see section “Investigating Neural Correlates of L1 Attrition”). As the process of attrition progresses, we may expect the attriters’ pattern of neural activation during L1 syntax processing to increasingly approximate that of low proficient AL-learners. Both groups, compared to L1 native speakers, are expected to exhibit stronger activity in Broca’s area, as well as activity in additional areas surrounding it.

## Future Directions

In view of international mobility and the rising number of bi- and multi-lingual communities, perhaps the first question to ask at the conclusion of this review concerns the nature of attrition as a separate field. To the extent that L1 attrition is caused by L2-related processes such as cross-language interference or transfer, attrition can be viewed as part of the general bilingual research highlighting the bidirectional cross-linguistic influence, as suggested by Kroll and colleagues (e.g., Bice and Kroll, 2015, 2019). Indeed, this review clearly demonstrates the substantial contribution of L2 related processes to attrition. However, it further suggests that L1-related processes contribute importantly to attrition, nonetheless. Thus, it seems that studying attrition as a separate field may extend the scope of our query and deepen our understanding of this phenomenon.

L1 attrition has been reported to affect all the main subsystems involved in language processing, with changes ranging from the lexico-semantic and phonological to the morphosyntactic levels of processing, in both the production and comprehension modalities. Despite its impact, the nature and the specific causes of the loss of native language capacity in attriting populations remain unclear. A number of factors have been implicated, including the amount and quality of L1 exposure, attitudes toward L1, and the cross-linguistic influence exerted by the L2. However, findings have so far provided mixed results. Above, we proposed two directions for future research that would advance our understanding of attrition phenomenon. The first direction offers an “audit” of two causal mechanisms hypothesized to drive attrition: one based on L1 disuse and the other on L2 transfer and interference. The second direction focuses on applying neuroscientific methods. As language is a highly dynamic phenomenon, it requires equally dynamic research techniques, such as neuroimaging tools with high temporal resolution (e.g., EEG/MEG) that would be sensitive to subtle modulations in language processing due to attrition (Shtyrov and Stroganova, 2015). Moreover, when applying such neuroscientific methods, it is important to use fine-grained experimental tasks that reflect the distinctive functions and representation in the complex array of linguistic process affected by attrition.

Importantly, in pursuing these research directions, it is imperative to carefully examine characteristics of the participants and obtain a highly detailed profile of the attriters’ L1 and L2 backgrounds, as many aspects of the language history and present patterns of use may affect attrition. Applying a theoretical framework in which L1 attrition is assumed to proceed as the reversal of L2 acquisition [see Kasparian and Steinhauer (2016, 2017) for extensive discussion of this idea] highlights the relevance of comparing L1 attriters to various groups L2 learners including AL-learners and bilingual minorities who are not immigrants, in addition to monolingual speakers. Another population that may provide interesting findings is attriters with no contact with the L2. Such attriters are rare, and often the lack of contact with L2 is related to background issues such as aging, low social status etc. Nevertheless, examining this group may clarify whether native language attrition is actually caused by cross-linguistic influence or by internal processes related to L1 reorganization. Recruiting balanced control groups is undoubtedly challenging in the context of attrition research. An alternative method is single-group longitudinal studies that test attriters repeatedly over several years (e.g., De Bot and Clyne, 1994; Hutz, 2004). Longitudinal studies may prove strongly informative about the time course of attrition and the factors that modulate it.

In summary, the present paper paves the way for a systematic investigation of the L1 attrition phenomenon. The review first outlined what is not attrition, then discussed central theoretic views regarding the origins of attrition and the methodological considerations that need to be adjusted to allow informative investigation of this phenomenon in future studies. Importantly, we focused on the key role of a neuroscientific approach in advancing a theoretical framework for the investigation of attrition. In particular, we highlighted the potential contribution of neuroimaging methods to reveal subtle modifications in language processing of attriters, which are not detectable by behavioral or self-report measures. Moreover, we believe that future studies that associate differences in the patterns of brain activation with differences between attriters, monolinguals and bilingual groups with different bilingual backgrounds (i.e., linguistic minorities and AL-learners) would promote a comprehensive theoretical framework that will portray attrition in relation to other bilingual processes such L2 learning and proficiency and cross-language processes. Finally, systematic neuroimaging examination of linguistic tasks of different representations (i.e., phonological, lexico-semantic, syntactic and pragmatic) and different functions (i.e., production, comprehension, reading) would reveal the neural correlates of specific aspects of L1 attrition and may shed light on the causal mechanisms of attrition and the different factors that influence these mechanisms. Hopefully, this future-oriented review will constitute a first step toward more detailed and more generalizable findings in this relatively young yet important research field.

## Author Contributions

FG, AM, and YS conceived the manuscript. FG and TC carried out extensive compilation and literature review. FG and BBM wrote the first draft of the manuscript. TC, HK, and AP wrote sections of the manuscript. FG, AM, YS, YA, and BBM reviewed the manuscript and contributed to the final version. AM supervised the project and received necessary funding. All authors contributed to manuscript, read, and approved the submitted version.

## Conflict of Interest

The authors declare that the research was conducted in the absence of any commercial or financial relationships that could be construed as a potential conflict of interest.

## Publisher’s Note

All claims expressed in this article are solely those of the authors and do not necessarily represent those of their affiliated organizations, or those of the publisher, the editors and the reviewers. Any product that may be evaluated in this article, or claim that may be made by its manufacturer, is not guaranteed or endorsed by the publisher.
